# The effect of walking on falls in older people: the 'Easy Steps to Health' randomized controlled trial study protocol

**DOI:** 10.1186/1471-2458-11-888

**Published:** 2011-11-24

**Authors:** Alexander Voukelatos, Dafna Merom, Chris Rissel, Cathie Sherrington, Wendy Watson, Karen Waller

**Affiliations:** 1 Health Promotion Service, Clinical Support Division (Western), Sydney, Australia; 2 School of Biomedical and Health Sciences, University of Western Sydney, Campbelltown, Australia; 3 School of Public Health, University of Sydney, Sydney, Australia; 4 The George Institute for Global Health, University of Sydney, Sydney, Australia; 5 Transport and Road Safety (TARS) Research, Department of Aviation (formerly NSW Injury Risk Management Research Centre), University of New South Wales, Sydney, Australia; 6 Health Promotion Service, Health Reform Transitional Organisation Southern, Sydney, Australia

## Abstract

**Background:**

Falls in older people continue to be a major public health issue in industrialized countries. Extensive research into falls prevention has identified exercise as a proven fall prevention strategy. However, despite over a decade of promoting physical activity, hospitalisation rates due to falls injuries in older people are still increasing. This could be because efforts to increase physical activity amongst older people have been unsuccessful, or the physical activity that older people engage in is insufficient and/or inappropriate. The majority of older people choose walking as their predominant form of exercise. While walking has been shown to lower the risk of many chronic diseases its role in falls prevention remains unclear. This paper outlines the methodology of a study whose aims are to determine: if a home-based walking intervention will reduce the falls rate among healthy but inactive community-dwelling older adults (65 + years) compared to no intervention (usual activity) and; whether such an intervention can improve risk factors for falls, such as balance, strength and reaction time.

**Methods/Design:**

This study uses a randomised controlled trial design.

A total of 484 older people exercising less than 120 minutes per week will be recruited through the community and health care referrals throughout Sydney and neighboring regions. All participants are randomised into either the self-managed walking program group or the health-education waiting list group using a block randomization scheme.

Outcome measures include prospective falls and falls injuries, quality of life, and physical activity levels. A subset of participants (n = 194) will also receive physical performance assessments comprising of tests of dynamic balance, strength, reaction time and lower limb functional status.

**Discussion:**

Certain types of physical activity can reduce the risk of falls. As walking is already the most popular physical activity amongst older people, if walking is shown to reduce falls the public health implications could be enormous. Conversely, if walking does not reduce falls in older people, or even puts older people at greater risk, then health resources targeting falls prevention need to be invested elsewhere.

**Trial Registration:**

Australia and New Zealand Clinical Trials Register (ANZCTR): ACTRN12610000380099

## Background

Falls in older people continue to be a major public health issue in industrialized countries [1]. Injuries resulting from falls are a leading cause of death and hospitalisation in people aged 65 years and over [2]. Falls can also lead to poor quality of life, loss of independence, and nursing home admission [3].

In New South Wales (NSW), the most populous state in Australia, there is already a heavy economic burden on the health system due to the cost of falls in older people. In 2006/07, the total lifetime cost of fall-related injury among older people in NSW was estimated at $558 million [4]. As in other industrialized countries and the rest of Australia, the NSW population is ageing and, based on demographic change alone, the cost to the health system of fall-related injuries is predicted to triple by 2050 [5].

Strategies to prevent falls among older people have been extensively researched over the past few decades resulting in a strong evidence-base for effective intervention in this area. Exercise is now recognized as a proven stand-alone fall prevention strategy [6,7] and is currently part of the evidenced-based recommendations in the UK-USA,[8] and Australia [9].

Despite over a decade of promoting physical activity, hospitalisation rates due to falls injuries in older people are still increasing [10,11]. This could be because efforts to increase physical activity amongst older people have been unsuccessful (or are unsuccessful amongst those most at risk of falling), or the physical activity that older people engage in is insufficient and/or inappropriate for falls prevention.

Various modalities of exercise have been tested in relation to their efficacy in preventing falls. In a recent meta-analysis of 54 physical activity-based trials studies it was found that most (64%) of the variability between trials was explained by three program-related features: a higher dose of exercise (≥ 50 hours over the entire trial period), the inclusion of balance challenging training (i.e., activity that moves the centre of mass while narrowing of the base of support and minimizing upper limb support), and not including a walking component in the program [7]. The latter finding raises an important public health issue given that the majority of older people choose walking as their predominant form of exercise [12,13].

Walking can substantially lower the risk of many chronic diseases and ameliorate the health care costs of this population [14] yet, given the above findings, its role in falls prevention remains unclear. One study found that people aged 75 years or older who were randomised to an individually prescribed home-based exercise program, which included walking, reported fewer falls than those randomised to a control group [15]. However, another study that examined the effects of walking on bone mass density in post-menopausal women found that participants in a brisk walking group had higher fall rates compared to the control group [16]. It is possible that increased walking by older adults results in greater exposure to the risk of falling (e.g., environmental hazards). This is consistent with studies that found the highest proportion of outdoor falls occur while walking [17,18].

Further, the potential of walking to improve risk factors of falls, such as balance, is also unclear. A 15-week walking program showed no significant effect on balance among sedentary, post-menopausal women [19]. On the other hand, a study which compared three different balance training programs, including walking, found that all three programs improved dynamic balance, but walking had a bigger impact on static balance [20].

Given the inconsistent evidence on the benefits of walking in the area of falls prevention, the aims of this study are to determine:

i. if a home-based walking intervention will reduce the falls rates among healthy but inactive community-dwelling older adults (65 + years) compared to no intervention (usual activity) and,

ii. whether such an intervention can improve risk factors for falls, such as balance, strength and reaction time.

## Methods

### Design

**Figure 1:**
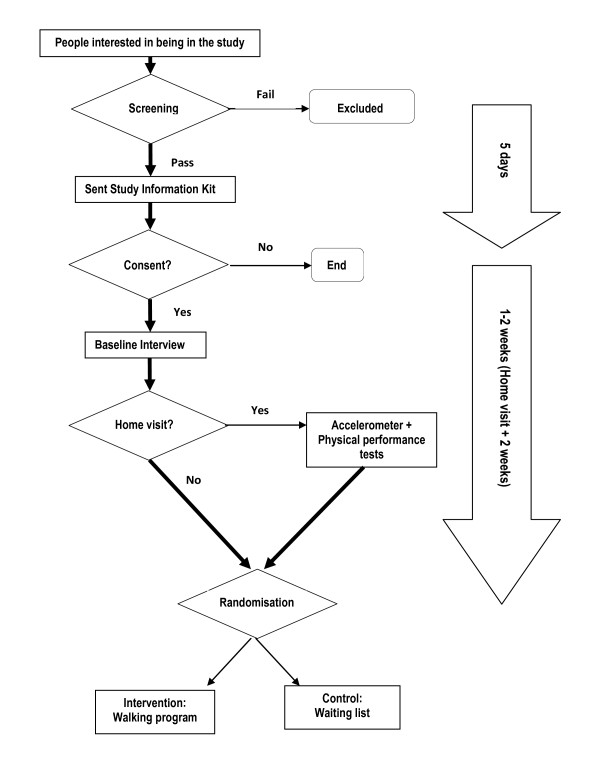
**Flowchart of participant recruitment**.

The design of this study is a randomized controlled trial (see Figure 1). Ethical approval to conduct this trial has been granted by the Research Ethics Review Committee of the Sydney South West Area Health Service-Eastern Zone (X08-0279 & HREC/08/RPAH/477). The study is registered with the Australian New Zealand Clinical Trials Registry (ACTRN12610000380099).

#### Participants

Sedentary, independent, community-dwelling people aged 65 years and over will be eligible to be recruited into this study. A person will be considered sedentary if they are participating, on average, in less than three moderate intensity sessions of physical activity or equivalent per week, totaling no more than 120 minutes [21]. Eligible participants will be also: able to speak and read English proficiently; walk unaided or with minimal assistance (e.g. walking stick) for at least 50 meters; free of any cognitive impairment or neurological conditions limiting their full participation in the study (e.g. dementia, debilitating arthritis, severe vision impairment); otherwise able to participate in physical activity or an exercise program unsupervised, and; not participating or enrolled in any other study.

#### Recruitment and randomization

Participants will be recruited using a variety of methods which include paid advertisements and editorials in community newspapers, use of elector information, distribution of flyers and other printed material promoting the study and through recommendations by study participants. Potential participants will call a research assistant who then screens them for eligibility as well as collecting basic demographic information. A study information kit, which includes a participant information sheet, an ethics consent form, and a medical clearance form (which asks participants to record any medical conditions and medications they are taking for these conditions), will then be posted. On completion and return of the consent and medical clearance forms a baseline interview will be conducted.

After completing the baseline interview participants will be asked if they would be willing to undergo a set of physical performance tests in their own home. Participants choosing not to be a part of the physical performance tests will be randomised immediately after the baseline interview is completed. The sub-set of participants agreeing to undergo physical performance tests will be randomised after the physical performance measures have been completed.

Participants will be randomised into either the self-managed walking group program or the health-education waiting list using sealed opaque envelopes prepared according to a block randomization scheme. The chief investigator (AV) developed the randomization scheme using a table of random numbers and will not be involved in recruitment or data collection. Participants will be assigned to groups after the baseline interview or, for a sub sample of participants, when the physical performance measures are completed. Participants will obviously not be blinded to group allocation. There will be no information about individual participant's group allocation revealed to research assistants during the follow-up interview until the last set of questions which are related to evaluation of the program.

#### Intervention

Participants randomised to the intervention group will receive a self-managed, progressive 'brisk' walking program. This consists of a series of three manuals and telephone coaching conducted over 12-months (48 weeks). The program will start at a level appropriate for sedentary individuals, and will be tailored to accommodate each individual's baseline physical activity levels. The program will progressively guide participants to increase their frequency and duration of walking until participants achieve a minimum of 150 minutes of 'brisk' walking (defined as walking at a pace where you can just barely hold a conversation while walking) per week (for example, over 5 days/week of 30 minute walks or more). This level of intensity is defined as a moderate walking pace which will maintain a safe yet beneficial level of activity for inactive older adults [19,22].

The walking manuals and the telephone support target five constructs from social cognitive theory. These constructs include: knowledge (i.e., walking for health benefits, walking recommendations, safety issues), behavioral skills (i.e., how to use pedometers for feedback, assessment of walking efforts), goal-directed behaviour and self-regulation (i.e., defining short-term achievable goals for each week and for each phase, recording of walks/steps), outcome expectations (i.e., recognizing personal benefits from the walking regimen) and reinforcement (i.e., rewards for reaching goals) [23,24]. The walking manuals have been designed to correspond to three intervention phases aimed towards building an exercise routine for sedentary adults.

**Figure 2:**
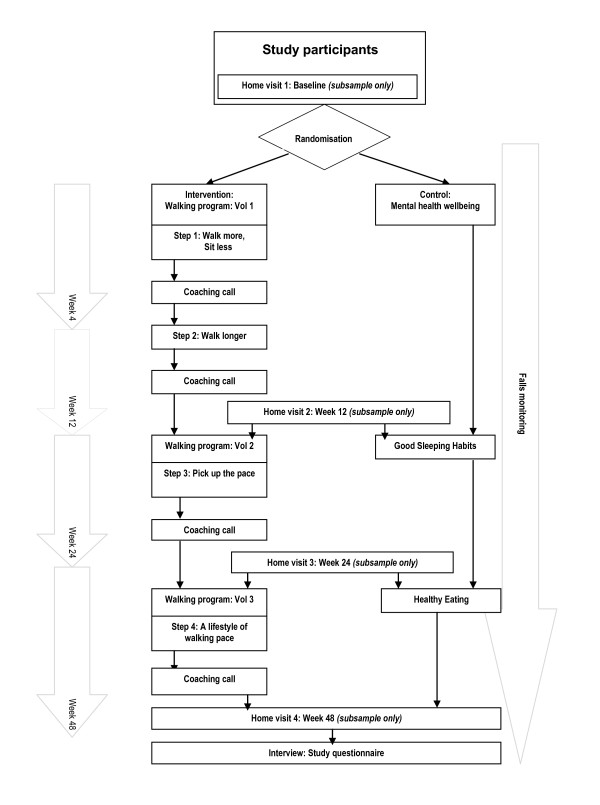
**Flowchart of participants through the study**.

The first phase of the walking manual (Walking Manual: Volume One) is the adoption phase, of 12 weeks duration, which focuses on accumulating walking time through a gradual increase of the frequency (weeks 1-4 of the program) and the duration (weeks 5-12) of walking according to the participants ability. Phase two (Walking Manual: Volume Two) is a transition phase (weeks 13-24), which focuses on increasing walking intensity to a brisk pace. The third phase (weeks 25-48) is the maintenance phase which focuses on strategies to maintain the level of walking reached in the previous phase [25]. The maintenance phase also includes strategies to manage setbacks and relapses. Participants receive telephone counseling at the beginning and mid-point for each phase of the program; a total of eight calls. Figure 2 is a flowchart of how participants progress through the study.

Intervention group participants will record the duration and frequency of walking, on their falls calendar. Although not a compulsory part of the program, these participants will also be encouraged to use a pedometer mainly as a motivational tool to encourage participants to keep walking. Participants can do this by comparing how many steps they have taken during their walking program towards a previously set goal. The pedometer used will be the Yamax Digiwalker SW-200 pedometer, which has been successfully used to promote walking in community dwelling adults [26].

The control group will receive health information over the duration of the study (48-weeks) covering health issues such as mental health wellbeing, healthy eating and developing good sleeping habits. Control group participants will be given no instruction on physical activity during the study, after which they also receive the walking kit. Control group participants will be sent a control group study kit consisting of a falls calendar and printed material on a health topic. Information on a new health topic will be mailed out at 12-weeks and 24-weeks in parallel with the intervention group. (See Figure 2)

#### Outcome measures

The main outcome measure is falls over the 12-month study period. A fall is defined as *'Unintentionally coming to rest on the ground, floor, or other lower level' *[27,28]. Falls will be recorded on a falls calendar, a method used successfully in previous falls prevention studies [27]. Each fall will be followed up with a telephone call by a research assistant to confirm the fall and to ascertain the circumstances around the fall including whether the fall resulted in any injuries, what treatment was sought.

Secondary outcome measures include:

• Dynamic balance and strength (sub-sample only)

• Physical performance (sub-sample only)

• Quality of life

• Walking behaviour

Other measures will be collected through the initial screening and baseline questionnaire and include basic demographic information, falls history, falls efficacy, self-reported levels of physical activity, walking self-efficacy and perceived neighbourhood walkability.

##### Prior to screening

###### Demographic characteristics

Demographic characteristics will be collected during initial screening, including country of birth, language spoken at home, highest level of education, current work situation, if any Government pensions/benefits are received, living situation, and total time spent in a car as a driver or passenger each week. In addition, data on any medical conditions and medication use will be collected through the medical clearance forms participants fill out prior to commencing the study (see Recruitment and Randomization).

###### Cognitive impairment

Participants' cognitive status will be determined at screening through the Short Portable Mental Status Questionnaire [29]. Respondents will be excluded from the study if they have more than three errors in the questionnaire indicating some level of cognitive impairment.

##### Study Questionnaire

The study questionnaire is designed to gather information on various factors.

###### Falls History and Falls Efficacy

Study participants will be asked to recall any falls over the past 12 months prior to being interviewed for the baseline questionnaire. Details on the number of falls, nature of fall, injuries sustained, perceived risk of falling and visits to a general practitioner will be recorded.

Confidence in avoiding a fall will be measured using the International Fear of Falling Questionnaire (FES-I). This 16-item questionnaire includes more complex activities relevant for less fragile older people when compared to the original FES questionnaire. It has been shown to have excellent reliability and construct validity [30].

###### Physical Activity

The Incidental and Planned Exercise Questionnaire (IPEQ) is a self-report questionnaire covering frequency and duration of several levels of planned and incidental physical activity in older people [31]. It assesses low, moderate and high levels of physical activities specifically designed for older people; the question and response categories accommodate both frail and active older adults ensuring the questionnaire is appropriate for use in clinical intervention trials, and it has good reliability and validity [31].

###### Quality of Life

Quality of life will be assessed using the Assessment of Quality of Life (AQoL) Mark 2. This tool comprises of 20 items selected from 6 dimensions of health: independent living, social and family, mental health, coping, pain, and sense perceptions [32]. This questionnaire has been developed based on the AQoL Mark 1 which has been shown to be a sensitive and valid measure of health-related quality of life (HRQL) in community-dwelling older people [33].

###### Walking Self-Efficacy

Walking self-efficacy will be assessed with two scales. The first scale measures behavioral self-efficacy [34]. Participants rate how confident they are in walking three days per week at a brisk pace for 10, 20, 30, 40, 50 and 60 minutes **c**ontinuously. The second scale assesses self-efficacy in regards to barriers [35]. Participants rate how confident they are to walk at least three days a week in a variety of situations. Both scales use a five-point Likert scale.

###### Neighbourhood Walkability

Neighbourhood walkability will be assessed using questions adapted from the abbreviated Neighbourhood Environment Walkability Scale (NEWS-A) [36]. This scale assesses perceived environmental attributes believed to influence physical activity and has been shown to have good reliability and validity [36,37].

##### Physical performance outcomes

The subsample of the study participants undergoing the physical performance assessments will have a home-visit arranged approximately two weeks after completing their baseline interview. Ten days before the scheduled home visit these participants will be sent an accelerometer, with detailed instructions on its use and the option of calling the research team for more information. On the testing day, the research assistant will conduct the physical performance measures with participants and collect the accelerometer.

The physical performance assessment has several components including: the Short Physical Performance Battery, quadriceps strength, lower limb reaction time, and physical activity levels. In addition participants' BMI will be measured. The physical performance assessments are undertaken in the privacy of participants' homes and are estimated to take between 20-30 minutes to complete.

###### Short Physical Performance Battery

The Short Physical Performance Battery (SPPB) measures lower limb functional status by assessing balance, gait, strength and endurance of the lower extremities. The tests include side-by-side, semi-tandem, and tandem standing tests, a timed walk, and sit-to-stand 5 repeat. The Short Physical Performance Battery is a standardized measure of lower extremity physical performance and is found to be efficient, practical, and safe to deliver in large cohorts of older adults [38,39].

###### Quadriceps Strength Test

Quadriceps strength will be assessed by seated leg extension of the dominant lower extremity, measured using a portable electronic dynamometer (Brand: CE. Model: OCS-2. Max = 60 kg, d = 20 g). The highest force achieved in three attempts is recorded [40].

###### Lower Limb Reaction Time

Lower limb reaction time will be measured through the Choice Step Reaction Time (CSRT). This test requires participants to perform quick, correctly targeted steps in response to visual cues [40]. This test has been identified as an independent and significant predictor of falls, and elucidates the roles of specific neuropsychological, sensorimotor, speed, and balance factors in the initiation of fast and appropriate step responses [41].

###### Physical Activity levels

Physical activity levels of the sub-sample of participants will be monitored using accelerometers (Actigraph GT1M, Pensacola FL USA). A sub-sample of participants, from both intervention and control groups, will be asked to wear accelerometers from the time they get out of bed until they go to sleep for 10 continuous days, timed to coincide with the physical performance measures at baseline, 3, 6 and 12-months.

###### Body mass index (BMI): Height and weight

Height will be measured in centimeters with a self-retracting construction measuring tape (max = 3 m, d = 0.5 cm). Weight will be measured in kilograms with digital weight scales (Propert Model: 3120. Max 150 kg, d = 100 g). BMI will be calculated from these measurements using the conventional formula BMI = Weight (kg)/(Height^2 ^(m^2^)).

#### Sample size

Previous falls prevention studies involving physical activity have shown a relative reduction of falls of between 20-35% [6]. Over a 12-month period it is expected that 33% of older people aged 65 years and older will fall at least once [6]. This study is powered at the alpha = 0.05 level with 80% power. A sample size of 232 per group is required to detect a relative reduction in falls of 35% (RR = 0.65) over 12-months. Adjusting the sample size to accommodate a 10% drop-out rate this study will need a sample size of 242 per group.

##### Sub-sample

Sample size calculation for the sub-sample is based on outcomes of the choice step reaction time test, which is a good approximation for overall dynamic balance [41]. Based on unpublished data from Central Sydney Tai Chi trial,[27] an estimated standard deviation of 0.2 ms can be expected. A sample size of 88 participants from each group will detect an absolute difference of 10% in reaction time. Assuming a 10% drop out rate, a sample size of 97 per group will be sufficient to detect a 10% absolute difference in reaction time between groups with an alpha of 0.05 and with power of 80%.

###### Analysis

All data will be analysed according to intention to treat analysis. Falls data will be compared across intervention and control groups at 12-months (i.e. 48-weeks) using a negative binomial regression model [27,28].

Outcome data obtained from the study questionnaire will be used to compare groups at 48-weeks. Falls efficacy, walking self-efficacy as well as perceived neighbourhood walkability will be analysed using the Kruskal Wallis test, a non-parametric test. Levels of physical activity and physical performance data will be analysed using regression models that will compare groups at 48-weeks using baseline values as a covariate.

A cost-effectiveness evaluation will calculate the cost of the walking program per fall prevented and a cost-utility analysis, based on the AQoL, will estimate the incremental cost-utility ratio expressed in terms of $AUD per QALY gain (or loss).

Sensitivity analyses will be conducted comparing unadjusted results and results adjusted for potential confounding variables. Potential confounding variables include age, gender, and previous falls. Data from the intervention group will also be analysed to investigate any dose-response relationships between level of walking and falls, and level of walking and physical performance.

## Discussion

Walking has been shown to have many health benefits, even later in life. For example, older men and women who walked regularly have reduced risk of all cause mortality, [42] of developing cardiovascular disease, [43] of cognitive decline, [44] and functional limitations [45]. Among postmenopausal women, adherence to walking regimens was associated with improved bone mass density,[46] favorable changes in blood lipids and glucose tolerance,[47] and reduced hypertension [48].

In a meta-analysis of exercise interventions to prevent falls, studies of exercise programs which included walking programs had lesser fall prevention effects than exercise programs that did not include walking programs. The authors suggest that the effect of walking may be cofounded by the tendency to prescribe walking regimens in trials involving high-risk population. Further, it was postulated that multi-component programs (e.g. balance, and walking) might be less efficient if time devoted to the most beneficial component, which is balance training, is compromised [7]. This study addresses these questions. It recruits community-dwelling older people not based on their falls risk, and it prescribes only walking with no other competing activity that can dilute or enhance the effect.

Despite evidence showing that certain types of physical activity reduce the risk of falls there still remain significant barriers for older people to become more active. In an effort to increase activity levels public health recommendations highlight the metabolic and cardiovascular benefits of accumulated daily day activity as long as it is of moderate-intensity. This includes household chores, gardening and walking. The role of lifestyle physical activity in relation to falls risk is under-studied. In this respect our study will be the first to determine if accumulated daily episodes of walking in older people's environments is also beneficial for falls.

Walking potentially overcomes many barriers for regular active lifestyle. It is already the most popular physical activity amongst older people requiring no training or special equipment. Most importantly, it is free. If walking is shown to reduce falls the public health implications could be enormous. Conversely, if walking does not reduce falls in older people, or even if it puts older people at greater risk then health resources targeting falls prevention would need to address those who choose walking as their single form of exercise, to increase their protection. This could be done by reducing falls hazards in the environment and by educating walkers of the need to maintain good balance before starting walking regimens and providing examples of simple home-based balance enhancing exercises.

## Competing interests

The authors declare that they have no competing interests.

## Authors' contributions

AV conceived of the study, lead the coordination of the study and the drafting of the manuscript, contributed to the design of the study and the development of the intervention. DM lead the development of the intervention, contributed to the design, coordination and drafting of the manuscript. CR, CS and WW all contributed to the design, and coordination of the study, the development of the intervention and the drafting of the manuscript. KW contributed to the coordination of the study, the development of the intervention, collection of data and the drafting of the manuscript. All authors have read and approved the final manuscript.

## Pre-publication history

The pre-publication history for this paper can be accessed here:

http://www.biomedcentral.com/1471-2458/11/888/prepub
